# Dexmedetomidine prevents cardiomyocytes from hypoxia/reoxygenation injury via modulating tetmethylcytosine dioxygenase 1-mediated DNA demethylation of Sirtuin1

**DOI:** 10.1080/21655979.2022.2054762

**Published:** 2022-04-07

**Authors:** Li Wang, Shaowei Wang, Tong Jia, Xiaojia Sun, Zhen Xing, Hui Liu, Jie Yao, Yanlin Chen

**Affiliations:** Department of Anesthesiology. First Affiliated Hospital of Hebei North College, Zhangjiakou, China

**Keywords:** Dexmedetomidine, cardiomyocyte, hypoxia/reoxygenation, TET1, DNA methylation, sirt1

## Abstract

Myocardial hypoxia/reoxygenation (H/R) injury is a common pathological change in patients with acute myocardial infarction undergoing reperfusion therapy. Dexmedetomidine (DEX) has been found to substantially improve ischemia-mediated cell damage. Here, we focus on probing the role and mechanism of DEX in ameliorating myocardial H/R injury. Oxygen–glucose deprivation and reoxygenation (OGD/R) were applied to construct the H/R injury model in human myocardial cell lines. After different concentrations of DEX’s treatment, cell counting kit-8 (CCK-8) assay and BrdU assay were employed to test cell viability. The profiles of apoptosis-related proteins Bcl2, Bax, Bad and Caspase3, 8, 9 were determined by Western blot (WB). The expression of inflammatory factors interleukin 1β (IL-1β) and tumor necrosis factor-α (TNF-α) was checked by reverse transcription-polymerase chain reaction (RT-PCR). By conducting WB, we examined the expression of NF-κB, Sirt1, Tet methylcytosine dioxygenase 1 (TET1) and DNA methylation-related proteins (DNA methyltransferase 1, DNMT1; DNA methyltransferase 3 alpha, DNMT3A; and DNA methyltransferase 3 beta, DNMT3B). Our data showed that OGD/R stimulation distinctly hampered the viability and elevated apoptosis and inflammatory factor expression in cardiomyocytes. DEX treatment notably impeded myocardial apoptosis and inflammation and enhanced cardiomyocyte viability. OGD/R enhanced total DNA methylation levels in cardiomyocytes, while DEX curbed DNA methylation. In terms of mechanism, inhibiting TET1 or Sirtuin1 (Sirt1) curbed the DEX-mediated myocardial protection. TET1 strengthened demethylation of the Sirt1 promoter and up-regulated Sirt1. DEX up-regulates Sirt1 by accelerating TET1 and mediating demethylation of the Sirt1 promoter and improves H/R-mediated myocardial injury.

## Graphical abstract

**Figure:**
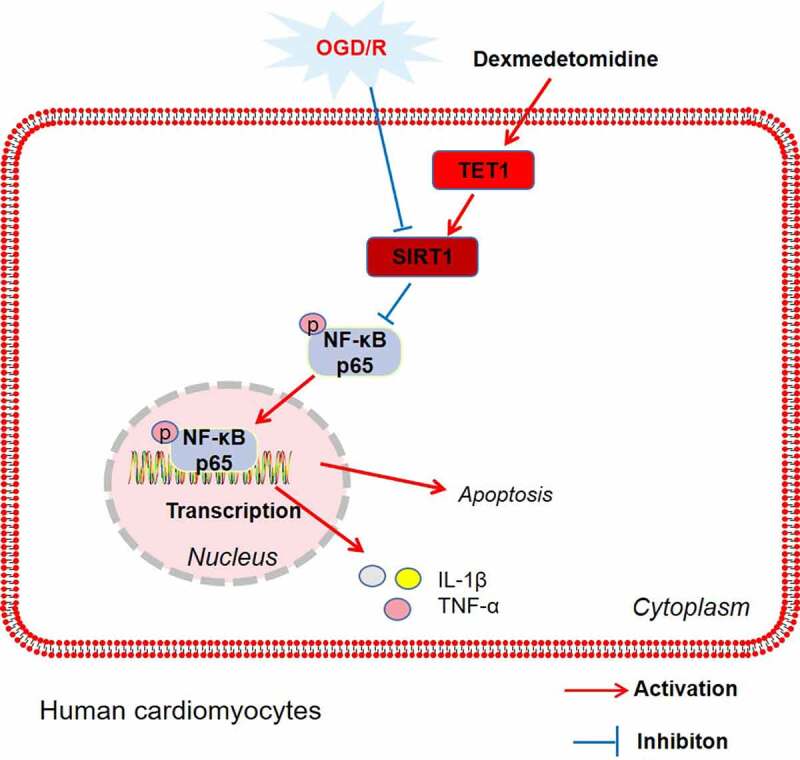

## 1. Introduction

Acute myocardial infarction (AMI) is myocardial necrosis induced by acute and persistent ischemia and hypoxia of the coronary arteries, which is associated with severe and persistent retrosternal pain clinically. Its incidence is rising year by year in China, seriously threatening human life and health [1]. Reperfusion therapy is the most vital and effective treatment for AMI, and reperfusion of myocardial blood can be achieved through pharmacological thrombolysis, stenting, or thrombectomy. Nevertheless, the restoration of blood supply usually activates a series of cellular injury mechanisms that may lead to myocardial cell death and an increase in infarct size, i.e., myocardial hypoxia/reoxygenation (H/R) damage [^2–4^]. As a result, it is essential to explore effective strategies to mitigate it.

Dexmedetomidine (DEX) is a highly selective α2-adrenergic agonist with analgesic, anti-stress, and anti-inflammatory effects and is commonly used as a sedative and anesthetic in clinical practice [5]. The protective effects of DEX in H/R-mediated organ injuries have been extensively studied, such as in brain [6], liver [7], renal [8]. Additionally, DEX exerts its role in cardiomyocyte through different mechanisms [^9–12^]. However, side effects, such as hypotension, gastrointestinal reaction, bradycardia, and tissue hypoxia, limit the application of Dexmedetomidine clinically [13]. Therefore, a closer look of the pharmacological, pharmacokinetic, and pharmacodynamic effects of dexmedetomidine is critical to maximize its safe, efficacious, and efficient pediatric perioperative applications [14].

Silencing information regulator 2 related enzyme 1 (Sirtuin1, Sirt1), also known as NAD-dependent deacetylase sirtuin-1, has important roles in tissue damage and repair, inflammation and autoimmunity [15]. Studies have displayed that Rutin eases H/R-induced cardiomyocyte injury via up-regulation of Sirt1 [16]. DEX curbs H/R injury-mediated apoptosis in cardiomyocytes by up-regulating Sirt1 via the Sirt1/CHOP pathway [17]. The nuclear factor kappa-B (NF-κB) is an essential intracellular transcription factor involved in modulating various biological functions, such as innate immunity, inflammation, cell proliferation, and apoptosis [18]. Numerous studies have exhibited that NF-κB activation is enhanced in H/R injury and that inhibition of NF-κB activation chokes the inflammation and apoptosis in cardiomyocytes [19,20]. Moreover, Sirt1 deacetylates NF-κB to hampers its transcriptional activity. As an example, Sirt1 overexpression impedes NF-κB acetylation, thereby improving cardiac function and reducing apoptosis in cardiomyocytes [21]. Tet methylcytosine dioxygenase 1 (TET1) is a demethylase that contributes to reducing DNA methylation levels and the expression of specific genes associated with inflammation [22]. DNA methylation, an epigenetic process catalyzed by DNA methyltransferases, is strongly linked to H/R injury [23]. Epigenetic analysis manifests that post-ischemic treatment curbs DNA hypomethylation of the DNA methyltransferase (DNMT) 3b-mediated microRNA-30a (miR-30a) promoter, protecting older cardiomyocytes from H/R injury through DNMT3b-dependent activation of miR-30a [24]. Also, gestational diabetes-mediated inhibition of Sirt1 is epigenetically regulated through DNA hypermethylation in the fetal heart and that inhibitors of DNA methylation enhance Sirt1 expression [25]. Accordingly, we speculated that TET1 could alleviate H/R damage, and there was some relationship between TET1 and Sirt1.

Here, we confirmed that DEX diminished oxygen–glucose deprivation and reoxygenation (OGD/R)-mediated myocardial injury, and DEX up-regulated Sirt1 and TET1, whereas restrained the activation of NF-κB. Thus, we hypothesized that DEX up-regulated Sirt1 by fostering TET1-mediated demethylation of the Sirt1 promoter, which ameliorated H/R-mediated myocardial injury. This study investigated the mechanism of TET1/Sirt1 in the amelioration of H/R injury by DEX, which is expected to provide fresh ideas and directions for the prevention and treatment of H/R injury.

## 2. Materials and methods

### 2.1 Cell culture

Human cardiomyocytes HCM and AC16 were bought from The Cell Center of the Chinese Academy of Sciences (Shanghai, China). Cells were grown in Dulbecco’s Modified Eagle’s medium (DMEM) comprising 10% fetal bovine serum (FBS) and 1% penicillin/streptomycin (Invitrogen, CA, USA) and incubated at 37°C with 5% CO_2_. The DMEM and FBS were obtained from Thermo Fisher Scientific (MA, USA). In the logarithmic growth phase of the cells, 0.25% trypsin (Thermo Fisher HyClone, Utah, USA) was applied for trypsinization and subculture [26].

### 2.2 Cell transfection and treatment

Before transfection, the cardiomyocytes were dispersed with 0.25% trypsin and inoculated into 6-well plates (1 × 10^5^ cells/well). When the cells grew to 80%–90% fusion, they were trypsinized and harvested. pcDNA 3.1 Sirt1 and the paired negative control (vector), small interfering RNA (si)-TET1 and Small interfering RNA negative control (si-NC), si-Sirt1 and the negative control (si-NC) were transfected into HCM and AC16 cells by adopting Lipofectamine 2000 (Invitrogen, Carlsbad, CA, USA). Cell transfection was performed according to a previous study [27]. HCM and AC16 cardiomyocytes were manipulated with DEX (5 μM) for 48 hours [17,28].

### 2.3 Oxygen-glucose deprivation and reoxygenation (OGD/R) model construction

In this study, an OGD/R model was constructed in human cardiomyocyte cell lines (HCM and AC16) to simulate myocardial H/R injury *in vitro*. The cardiomyocytes were cleaned with phosphate-buffered saline (PBS) three times, and the medium was substituted with a glucose-free DMEM. The *in-vitro* OGD/R model was constructed by the following steps: 1) the cells were cultured in a 37°C incubator with normal conditions of 95% air and 5% CO_2_ for 12 h; 2) the cells were put in an anaerobic system (MIC-101, Labcompare, SanFrancisco, USA) under the oxygen defcient (hypoxia) conditions of 1% O_2_, 94% N_2_ and 5% CO_2_ in the 37°C incubator for 12 h; 3) the cells were transferred into the normal conditions of 95% air and 5% CO_2_ for reoxygenation for 6 h[29]. Afterward, the glucose-free medium was replaced by a normal medium, and the cells taken from the anoxic chamber were cultured in normoxic conditions at 37°C with 5% CO_2_ for 24–72 hours. The cardiomyocytes were divided into the CON, DEX (5 µM), OGD/R, OGD/R + DEX (0.5 μM), OGD/R + DEX (1 μM) and OGD/R + DEX (5 μM) groups. Cardiomyocytes in the OGD/R and OGD/R + DEX (0.5, 1, 5 μM) groups were cultured in a hypoxic chamber, followed by reoxygenation under normoxic conditions. In contrast, the cells in CON and DEX (5 µM) groups were incubated under normoxic conditions at all times.

### 2.4 Cell counting kit-8 (CCK-8) experiment

Cardiomyocyte viability was assessed with the CCK-8 assay. Cardiomyocytes were inoculated into 96-well plates (1 × 10^3^ cells/well) and incubated for 24 hours. For the cell viability test, 10 μL of CCK-8 reagent (DojindoMolecularTechnologies, Kumamoto, Japan) was added to each well according to the manufacturer’s instructions and incubated at 37°C for at least one hour. The optical density (OD) 450 values were then observed utilizing a spectrophotometer (Bio-Rad, CA, USA) [30].

### 2.5 BrdU experiment

HCM and AC16 cells at the logarithmic growth stage were made into single-cell suspensions and inoculated on 24-well plates at 1 × 10^5^ cells per well. After cell adherent growth, the BrdU labeling reagent was added following the sigma’s operation instructions, and the plates were incubated with 5% CO_2_ at 37°C. After 48 hours of continuous culture, immunofluorescence staining of cells was carried out as per sigma’s BrdU antibody operating instructions [31].

### 2.6 Reverse transcription-polymerase chain reaction (RT-PCR)

With the use of the TRIzol reagent (Invitrogen, Carlsbad, CA, USA), total RNA was extracted out of cells. The RNA was reverse-transcribed into cDNA by employing the PrimeScript™ RT Reagent kit (Invitrogen, Shanghai, China) by observing the manufacturer’s guidelines. qPCR was performed with a Bio-Rad CFX96 qPCR system and SYBR (SYBR Green qPCR Master Mix, MedChemExpress, NJ, USA) following the manufacturer’s protocols. PCR was carried out with pre-denaturation at 95°C for 5 min, followed by denaturation at 95°C for 15s and annealing at 60°C for 30s. Glyceraldehyde-3-phosphate dehydrogenase (GAPDH) served as an internal reference to check the expression of interleukin 1β (IL-1β) and tumor necrosis factor-α (TNF-α), which were calculated using the 2^−ΔΔCT^ method. Each test was repeated three times [32]. The primer sequences were as follows: IL-1β: forward 5’-CCGTGGACCTTCCAGGATGA-3’, reverse 5’-GGGAAGGTCACACACCAGCA-3’; TNF-α: forward 5’-CATCTTCTCAAAATTCGAGTGACAA-3’, reverse 5’- TGGGAGTAGACAAGGTACAACCC-3’. GAPDH: forward 5’-GGAGCGAGATCCCTCCAAAAT-3’, reverse 5’-GGCTGTTGTCATACTTCTCATGG-3’.

### 2.7 Western blot (WB)

After cell treatment, the medium was discarded. The protein lysate (Roche) was added, and the total proteins were isolated. 50 g of total protein was loaded on 12% polyacrylamide gel and electrophoresed at 100 V for two hours. It was then electrically transferred to polyvinylidene fluoride (PVDF) membranes (Millipore, Bedford, MA, USA). After being blocked with 5% skimmed milk powder for one hour at room temperature (RT), the membranes were cleaned three times with tris-buffered saline with Tween-20 (TBST) for 10 min each. Next, they were incubated with the primary antibodies (1:1000) of Sirt1 (Abcam, ab32441, Shanghai, China), anti-NF-κB (Abcam, ab32360), anti-p-NF-κB (Abcam, ab76302), anti-NF-κB p65 (acetyl K310) (ab19870), anti-Bcl2 (Abcam, ab32124), anti-Bax (Abcam, ab32503), anti-Bad (Abcam, ab32445), anti-C-Caspase3 (Abcam, ab32042), anti-C-Caspase8 (Thermo Fisher Scientific, MA5-38,680, Massachusetts, USA), anti-C-Caspase9 (Abcam, ab2324, Shanghai, China), anti-TET1 (Abcam, ab191698), anti-DNMT1 (Abcam, ab19905), anti-DNMT3A (Abcam, ab2850), anti-DNMT3B (Abcam, ab2851), and anti-GAPDH (Abcam, ab9485) overnight at 4°C. After washing the membranes with TBST, we incubated them with horseradish peroxidase (HRP)-labeled anti-rabbit secondary antibody (1:300) at RT for one hour, followed by three washes with TBST (10 min each time). Finally, the Western blot reagent (Invitrogen company) was applied for color imaging, and each protein’s gray value was analyzed via ImageJ [33].

### 2.8 Enzyme-linked immunosorbent assay (ELISA)

The 5-methylcytosine (5-mC) levels of genomic DNA were measured with a 5-mC DNA ELISA kit (colorimetric assay) (Zymo Research, Irvine, CA, USA) by observing the manufacturer’s instructions. Overall DNA methylation (expressed as % 5-MC) [34] was analyzed and absorbance was reviewed at 405 nm (Biotek, Elx800-Winooski, VT, USA).

### 2.9 Methylation-sensitive polymerase chain reaction (MS-PCR)

Genomic DNA from HCM and AC16 was extracted with the TIANampGenomicDNA Kit (TiangenBiotech, Beijing, China) and transformed by bisulfite using the EpiTectBisulfiteKit (Qiagen, Duesseldorf, Germany). MS-PCR was conducted with MS kits (TiangenBiotech) on purified DNA using both methylated and unmethylated primers following the manufacturer’s guidelines. The obtained products were visualized by agarose gel electrophoresis and GelRed (Vimed, Xzhou, China). All experiments were carried out in triplicate [35].

### 2.10 Establishment of animal I/R models

Sprague-Dawley rats weighing 220–250 g at 6–8 weeks of age were obtained from the Experimental Animal Center of Hebei Medical University [Approval number: SCXK (Ji) 2020–001]. The rats were exposed to standard rodent food and water in a controlled environment (room temperature 22°C, light/dark cycle for 12 hours). All animal experiments were granted by the Animal Experimentation Ethics Committee of First Affiliated Hospital of Hebei North College (2021–028), and experimental procedures were in accordance with the Guide for the Care and Use of Laboratory Animals (National Institutes of Health).

The rats were randomized into Sham, DEX, I/R, I/R + DEX and si-TET1 + I/R + DEX groups, with 10 rats in each group. The rats were administered si-TET1 lentivirus via tail vein prior to surgery. Before being induced ischemia, the rats received dexmedetomidine (a load dosage of 3 μg/kg at 30 min and then 3 μg/kg/min for 2 h) treatment [36]. Rats were anesthetized by intraperitoneal administration of 40 mg/kg sodium pentobarbital, intubated, and ventilated (tidal volume of 5 ml/kg; 80 breaths/min). The rats then underwent left thoracotomy and ligation of the left anterior descending (LAD) artery, which was approximately 2 mm from the left auricle. The LAD ligation was maintained for 30 minutes and then the ligature was released for 24 hours of blood reperfusion. The model establishment was confirmed by S-T segment elevation and myocardial thermal scalding. Throughout the procedure, the rats’ body temperature was maintained at 37°C by using a heating pad. After surgery, the myocardial tissues were harvested from rats for subsequent testing. At last, the animals were executed by intraperitoneal injection of 100 mg/kg sodium pentobarbital [37].

### 2.11 Hematoxylin-eosin (HE) staining

Rat myocardial tissues were taken and immobilized in 10% formaldehyde solution for 24 hours. After dehydration in gradient alcohol, the tissues were paraffin-embedded and cut into 4-μM sections. Following dewaxing and hydration, the myocardial tissues were stained with hematoxylin and eosin and examined for pathological alterations under a light microscope (Leica Microsystems, Wetzlar, Germany) [38].

### 2.12 TdT-mediated dUTP nick end labeling (TUNEL) assay

Paraffin-embedded myocardial tissue sections (4 μM) were dewaxed in xylene, dehydrated in graded alcohol and treated with proteinase K (10 mmol/L) for 15–30 minutes at 21–37°C. Next, the sections were flushed twice in PBS, immersed in the closure solution and closed at room temperature for 10 minutes. 50 μL of TdT enzyme reaction solution was added to the sections, which were covered with coverslips and maintained under a wet environment for 60 minutes at 37°C away from light. The water around the sample was dried, and 50 μL of converter-peroxidase (POD) was added and incubated in a wet box at 37°C for 20–30 minutes. Afterward, the sections were added to 50 μL of DAB substrate solution and left to incubate for 10 minutes at room temperature. At last, the sections were sealed with glycerol, and a light microscope was employed for counting and photographing [39].

### 2.13 Statistics and analysis

Statistical software SPSS 17.0 (SPSS Inc, Chicago, IL, USA) was adopted to analyze all experimental data in this study. Data were expressed as mean ± standard deviation (x ± S). Differences between multiple groups were compared using one-way ANOVA, and *t* test was employed for comparing differences between the two groups, with *P* < 0.05 being a statistically significant difference [40].

## 3. Results

OGD/R-mediated myocardial injury model was constructed, and DEX was administered. The viability, apoptosis, and inflammatory response were detected. Then, TET1 and Sirt1/NF-κB pathway were examined for investigating the mechanism of DEX in relieving myocardial injury. TET1 knockdown model was established in cardiomyocytes using si-TET1 for confirming the role of DEX in mediating TET1. Furthermore, an in-vivo myocardial injury model DEX was treated with

### 3.1 DEX curbed OGD/R-mediated myocardial injury

**Figure 1.**
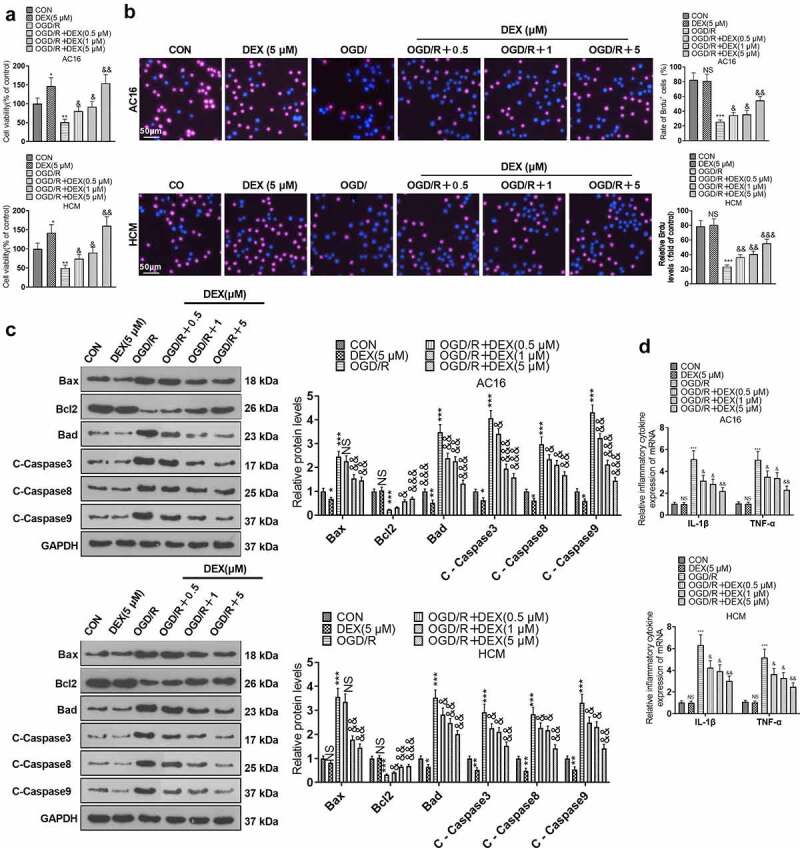
DEX inhibited OGD/R – mediated myocardial injury.

To ensure consistency of results, we checked the influence of different OGD durations on the viability of cardiomyocytes (AC16 and HCM). The CCK8 assay revealed a gradual decrease in cardiomyocyte viability following 1 to 4 hours of OGD treatment. However, there was no significant difference in the magnitude of the decrease in cardiomyocyte viability at the third and fourth hours of OGD (Figure 1a). Therefore, we chose to reoxygenate cardiomyocytes at 4 hours after OGD and evaluated the impact of various reoxygenation periods on cardiomyocyte viability. As a result, both 12 and 24 hours of reoxygenation attenuated cardiomyocyte viability compared to the CON group. In contrast, the cardiomyocyte viability was not substantially altered after 48 hours and 72 hours of reoxygenation. In parallel, DEX (1 μm) enhanced cardiomyocyte viability at both 12 and 24 hours of reoxygenation after 4 hours of OGD, but it had no remarkable impact on cardiomyocyte viability at 48 and 72 hours of reoxygenation (Figure 1b). Accordingly, we performed OGD for 4 hours and reoxygenation for 24 hours for the next experiments. To document the contribution of DEX in myocardial H/R injury, we treated AC16 and HCM cells with OGD for 4 hours and reoxygenation for 24 hours and then processed OGD/R-induced cardiomyocytes with DEX (0.5, 1, and 5 μM) for 48 hours to gauge proliferation, apoptosis, and inflammation in cardiomyocytes. The viability of HCM and AC16 was evaluated by CCK-8 and BrdU. By contrast with the CON group, OGD/R abated cardiomyocyte viability, while DEX (5 μM) boosted cardiomyocyte viability. DEX enhanced cardiomyocyte viability under OGD/R stimulation dose-dependently (vs. the OGD/R group) (Figure 1c, d). As indicated by WB results, by contrast with the CON group, OGD/R elevated the expression of pro-apoptotic proteins (Bax, Bad, Caspase3, Caspase8, and Caspase9) and hindered the expression of the anti-apoptotic protein (Bcl2) in cardiomyocytes. DEX (5 μM) down-regulated pro-apoptotic proteins in cardiomyocytes, while it had no major influence on the Bcl2 expression. In contrast, pro-apoptotic markers were abated and the anti-apoptotic marker was boosted in DEX-treated cardiomyocytes dose-dependently (vs. the OGD/R group) (Figure 1e). Moreover, the profiles of inflammatory factors TNF-α and IL-1β were heightened in OGD/R-induced cardiomyocytes (vs. the CON group), and there was little difference in the expression of inflammatory factors between the DEX and CON groups, as evidenced by RT-PCR. Besides, DEX dose-dependently curbed the levels of inflammatory factors in OGD/R-induced cardiomyocytes in comparison to the OGD/R group (figure 1f). These outcomes hinted that DEX choked OGD/R-mediated myocardial injury dose-dependently.

OGD/R-induced cardiomyocytes (AC16 and HCM) were manipulated with varying concentrations (0.5, 1, and 5 μM) of DEX for 48 hours. A: The viability of each group of cardiomyocytes (AC16 and HCM) was assayed with CCK-8. B: Detection of the proliferation in AC16 and HCM was made using BrdU. C: The profiles of apoptosis-related proteins (Bax, Bcl2, Bad and Caspase3, Caspase8, Caspase9) in AC16 and HCM were determined by WB. D. RT-PCR was conducted to verify the expression of inflammatory factors (TNF-α and IL-1β) in AC16 and HCM. Data were expressed as mean ±SD, n = 3; NS *P* > 0.05, *P < 0.05, **P < 0.01, ***P < 0.001, ****P < 0.0001 (vs. CON). &*P* < 0.05, &&*P* < 0.01, &&&*P* < 0.001 (vs. OGD/R).

### 3.2 DEX boosted Sirt1 and weakened NF-κB activation

**Figure 2.**
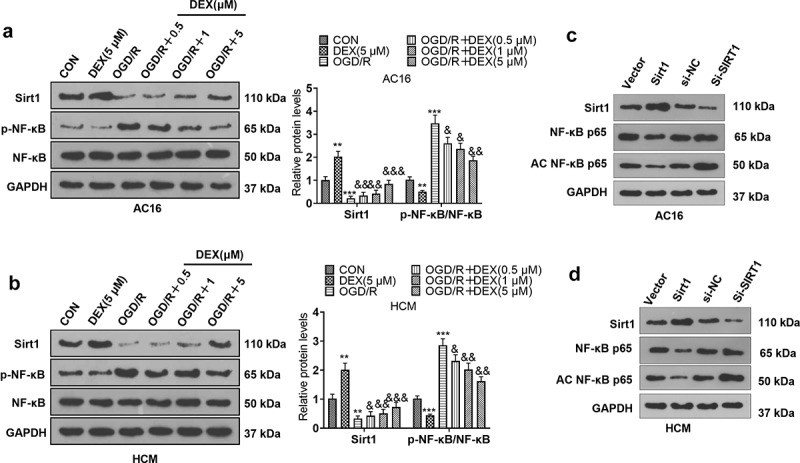
DEX facilitated Sirt1 and inactivated NF-κB.

To specify the action of DEX on Sirt1 and NF-κB, we assayed the expression of Sirt1 and NF-κB in cardiomyocytes (HCM and AC16) using WB. The results displayed that by contrast with the CON group, the OGD/R had restrained Sirt1 expression and lifted NF-κB phosphorylation. In comparison to the OGD/R group, DEX (0.5 to 5 μM) dose-dependently facilitated Sirt1 expression and inactivated NF-κB in OGD/R-induced cardiomyocytes. In addition, DEX enhanced Sirt1 expression and curbed NF-κB phosphorylation in cardiomyocytes (Figure 2a-b). We constructed Srit1 overexpression and downregulation model in cardiomyocytes. Then, we found that overexpression of SIRT1 inhibited the expression of NF-κB p65 and acetylated NF-κB p65. By contrast, knocking down SIRT1 enhanced the acetylation level of NF-κB p65 in cardiomyocytes (Figure 2c-d), indicating that SIRT1 regulates cellular functions through NF-κB deacetylation. These data confirmed that DEX elevated Sirt1 expression and inactivated the NF-κB pathway.

OGD/R-induced cardiomyocytes (AC16 and HCM) were processed with different concentrations (0.5, 1, and 5 μM) of DEX for 48 hours. A-B: Expression of Sirt1 and NF-κB in AC16 and HCM was assessed by WB. C-D. WB was carried out for detecting the protein expression of Sirt1, NF-κB p65, and AC NF-κB p65 in AC16 and HCM cells transfected with Sirt1 overexpression plasmids or si-Sirt1. Data were expressed as mean ±SD, n = 3; **P < 0.01, ***P < 0.001(vs.CON). &*P* < 0.05, &&*P* < 0.01, &&&*P* < 0.001 (vs. OGD/R).

### 3.3 Inhibiting Sirt1 weakened DEX-mediated myocardial protective effects

**Figure 3.**
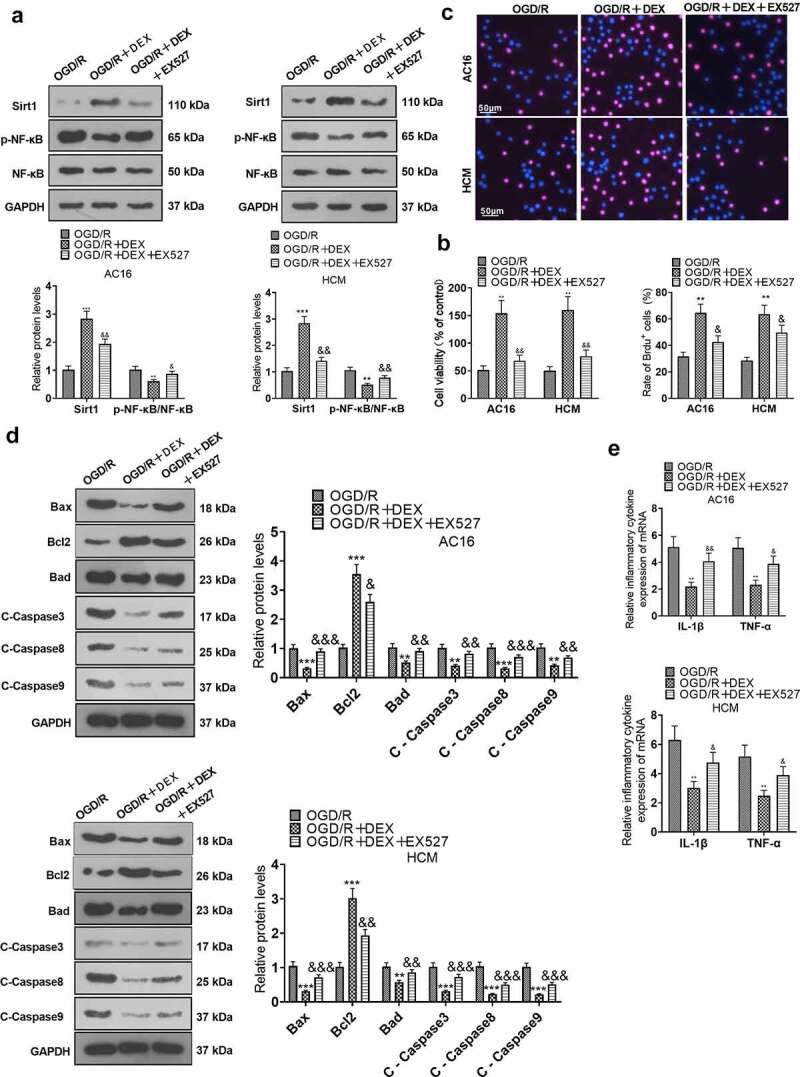
Inhibiting Sirt1 attenuated DEX-mediated myocardial protection.

To elucidate the function of Sirt1 in the amelioration of OGD/R injury in cardiomyocytes by DEX, OGD/R-induced cardiomyocytes were processed with 5 μM DEX for 48 hours, followed by the addition of a Sirt1 inhibitor (EX527). Expression levels of Sirt1 and NF-κB in HCM and AC16 cells were tested with WB. The data indicated that Sirt1 was up-regulated and the phosphorylation of NF-κB was attenuated in cardiomyocytes of the OGD/R + DEX group versus the OGD/R group. In contrast, the EX527 treatment repressed Sirt1 expression and enhanced NF-κB phosphorylation in cardiomyocytes versus the OGD/R + DEX group (Figure 3a). As uncovered by CCK-8 and BrdU assays, EX527 impaired OGD/R-induced cardiomyocyte viability versus the OGD/R + DEX group (Figure 3b-c). By utilizing WB, we discovered that the OGD/R + DEX (5 µM) + EX527 group exhibited higher expression of pro-apoptotic proteins and lower expression of anti-apoptotic proteins than that of the OGD/R + DEX (5 µM) group (Figure 3d). The levels of inflammatory factors IL-1β and TNF-α were measured by RT-PCR. It turned out that the inflammatory factor expression was higher in the OGD/R + DEX (5 µM) + EX527 group than that in the OGD/R + DEX (5 µM) group (Figure 3e). These data testified that dampening Sirt1 weakened the myocardial protective effect mediated by DEX.

OGD/R-induced cardiomyocytes (AC16 and HCM) were treated with 5 μM DEX for 48 hours, followed by the addition of a Sirt1 inhibitor (EX527). A: Expression of Sirt1 and NF-κB in AC16 and HCM was determined by WB. B: Detection of cardiomyocyte viability was performed in AC16 and HCM using CCK-8 assay. C: Cardiomyocyte proliferation was checked by the BrdU assay. D: Expression of apoptotic proteins (Bax, Bcl2, Bad and Caspase3, Caspase8, and Caspase9) in AC16 and HCM was monitored by WB. E. RT-PCR was conducted to measure the expression of inflammatory factors (TNF-α and IL-1β) in AC16 and HCM. Data were expressed as mean ±SD, n = 3; **P < 0.01, ***P < 0.001 (vs. OGD/R). &*P* < 0.05, &&*P* < 0.01, &&&*P* < 0.001 (vs. OGD/R+ DEX).

### 3.4 DEX expedited TET1 and suppressed OGD/R-mediated DNA methylation in cardiomyocytes

**Figure 4.**
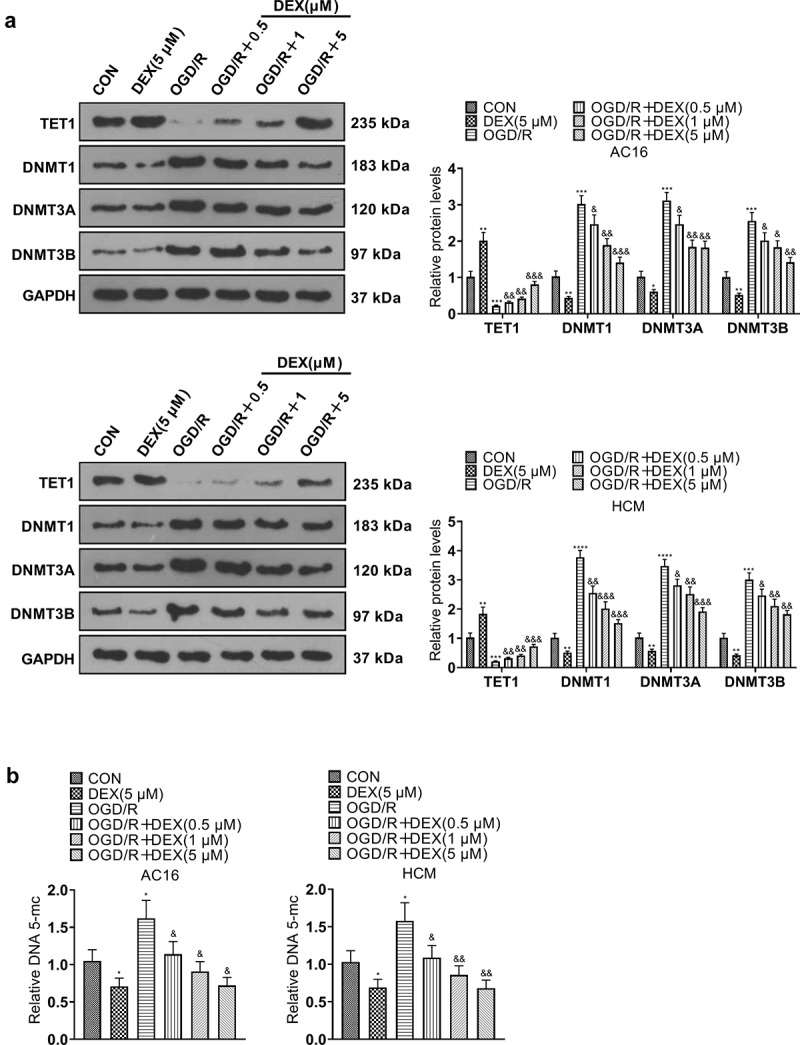
DEX boosted TET1 and abated OGD/R-mediated DNA methylation in cardiomyocytes.

To study the influence of DEX on TET1 expression and OGD/R-mediated DNA methylation in cardiomyocytes, we examined the expression of TET1, DNA methylation-related proteins and DNA methylation levels in cardiomyocytes (HCM and AC16). The profiles of TET1 and DNA methylation-related proteins (DNMT1, DNMT3A, and DNMT3B) in HCM and AC16 were measured by WB. The results disclosed that the TET1 expression was hindered and the levels of DNA methylation-related proteins were heightened in the OGD/R group (vs. the CON group). 5 μM DEX up-regulated TET1 and decreased the expression of DNA methylation-related proteins in cardiomyocytes. Besides, in comparison to the OGD/R group, DEX concentration-dependently lifted TET1 expression and hindered DNA methylation-related protein expression in cardiomyocytes (Figure 4a). The 5-mC DNA ELISA Kit exhibited facilitated DNA methylation in the OGD/R group versus the CON group. In contrast, 5 μM DEX declined DNA methylation levels in cardiomyocytes. DEX concentration-dependently diminished DNA methylation levels in OGD/R-induced cardiomyocytes compared to the OGD/R group (Figure 4b). In conclusion, DEX boosted the TET1 expression and reduced DNA methylation in OGD/R-mediated cardiomyocytes.

OGD/R-induced cardiomyocytes (AC16 and HCM) were processed with varying concentrations (0.5, 1, and 5 μM) of DEX for 48 hours. A: Expression of TET1 and DNA methylation-related proteins (DNMT1, DNMT3A, and DNMT3B) in AC16 and HCM was tested by WB. B: DNA methylation levels in AC16 and HCM were measured by adopting the 5-mC DNA ELISA Kit. Data were expressed as mean ±SD, n = 3; *P < 0.05, **P < 0.01, ***P < 0.001, ****P < 0.0001 (vs. CON). &*P* < 0.05, &&*P* < 0.01, &&&*P* < 0.001 (vs. OGD/R), &&*P* < 0.01.

### 3.5 TET1 knockdown curbed the DEX-mediated myocardial protective effects

**Figure 5.**
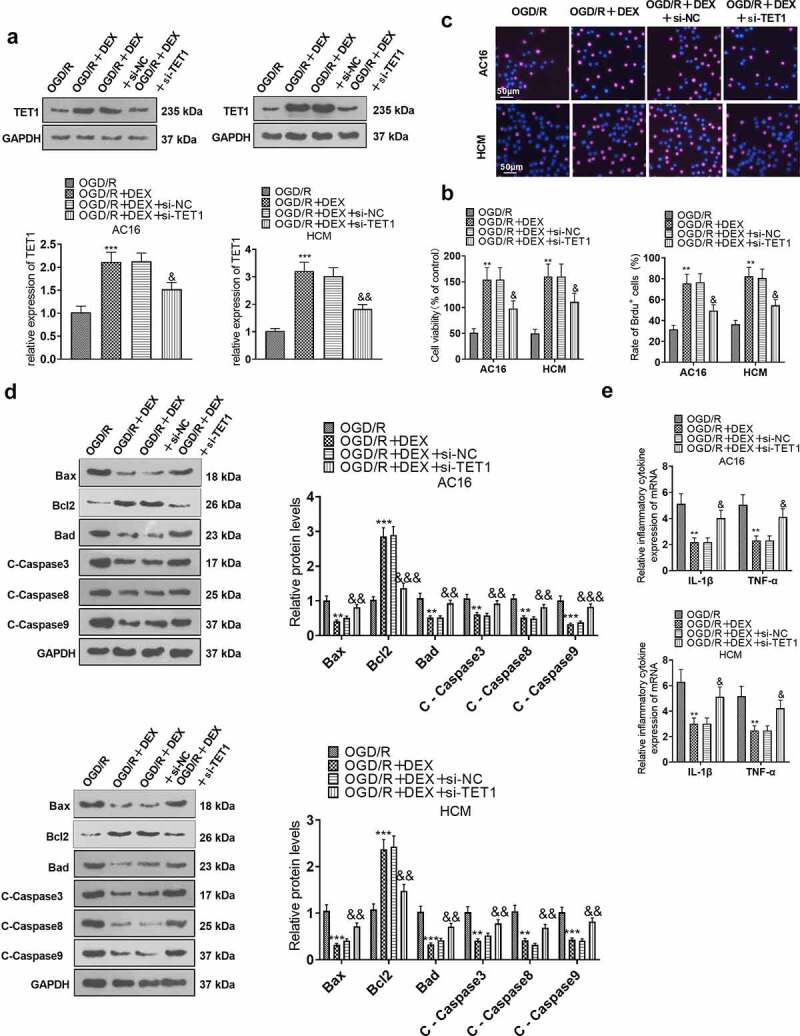
TET1 knockdown impeded the DEX-mediated myocardial protective effect.

To enquire into the action of TET1 in the amelioration of OGD/R injury in cardiomyocytes by DEX, si-NC and si-TET1 were transfected into cardiomyocytes (HCM and AC16). The transfected OGD/R-induced HCM and AC16 were then treated with 5 μM DEX for 48 hours to assay proliferation, apoptosis and inflammation. WB outcomes manifested that TET1 expression was curbed in cardiomyocytes transfected with si-TET1 (versus the OGD/R+ DEX+si-NC group, Figure 5a). CCK-8, BrdU and WB assays illustrated that cardiomyocytes in the OGD/R + DEX + si-TET1 group had intensified apoptosis and abated viability versus the OGD/R + DEX + si-NC group (Figure 5b, c, d). As confirmed by RT-PCR data, the expression of inflammatory factors was augmented in cardiomyocytes in the OGD/R + DEX (5 µM) + si-TET1 group compared to that of the OGD/R + DEX (5 µM) + si-NC group (Figure 5e). These results validated that DEX hampered OGD/R-mediated myocardial injury by elevating TET1 expression, and inhibiting TET1 attenuated the DEX-mediated cardioprotective effect.

The si-NC and si-TET1 were transfected into cardiomyocytes (HCM and AC16), and the transfected OGD/R-induced cardiomyocytes were then treated with 5 μM DEX for 48 hours. A: TET1 expression in AC16 and HCM was checked by WB. B: The viability of AC16 and HCM was tested using CCK-8. C: The BrdU assay was conducted to examine the proliferation of AC16 and HCM. D: The profiles of Bax, Bcl2, Bad, Caspase3, Caspase8, and Caspase9 in AC16 and HCM were determined by WB. E. The profiles of inflammatory factors (TNF-α and IL-1β) in AC16 and HCM were evaluated by RT-PCR. Data were expressed as mean ±SD, n = 3; **P < 0.01, ***P < 0.001 (vs. OGD/R). &*P* < 0.05, &&*P* < 0.01, &&&*P* < 0.001 (vs. OGD/R+ DEX+si-NC).

### 3.6 TET1 mediated the demethylation of the Sirt1 promoter and heightened Sirt1 expression

**Figure 6.**
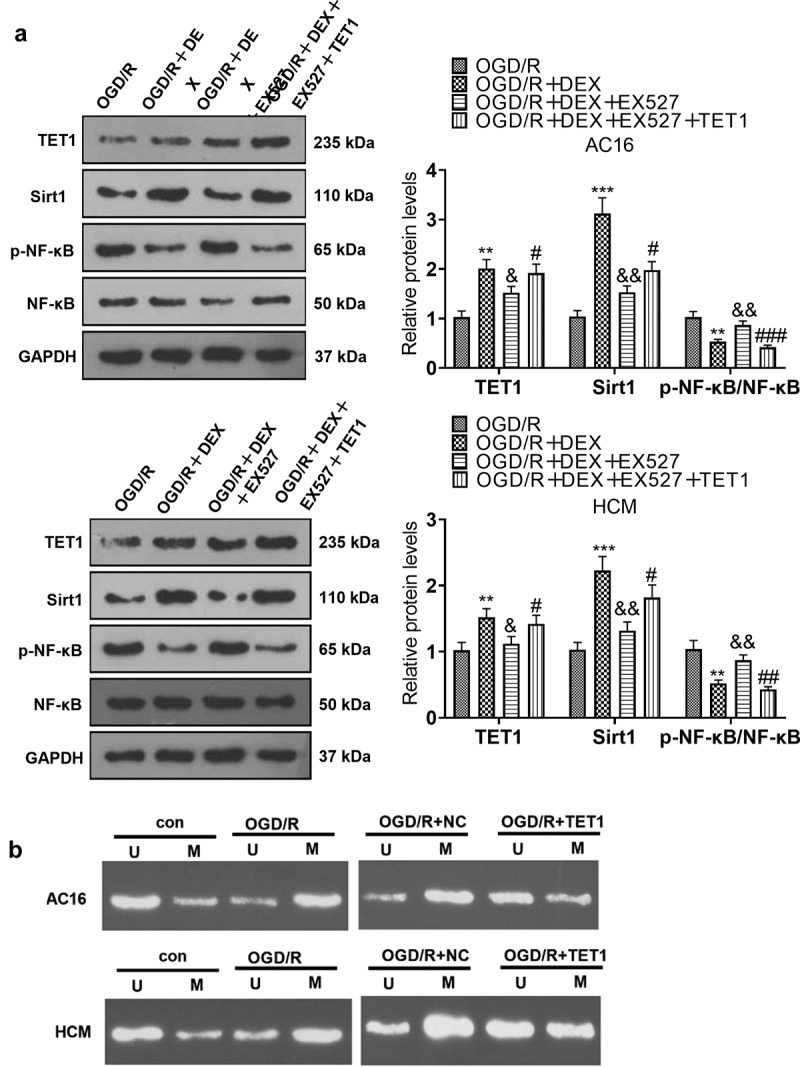
TET1 mediated demethylation of the Sirt1 promoter and lifted Sirt1 expression.

To characterize the influence of TET1 on Sirt1 expression and demethylation levels of the Sirt1 promoter, TET1 overexpression plasmids were transfected into cardiomyocytes (HCM and AC16). OGD/R-induced HCM and AC16 were then treated with 5 μM DEX for 48 hours and a Sirt1 inhibitor (EX527) was added to test cardiomyocyte viability, apoptosis, and inflammation. WB data uncovered that the OGD/R + DEX group displayed higher expression of Sirt1 than that of the OGD/R group, and the addition of EX527 to OGD/R + DEX (5 µM) hindered the Sirt1 profile, enhanced p-NF-κB level, but had no significant effects on TET1 expression. Meanwhile, the Sirt1 expression in the OGD/R + DEX + EX527 + TET1 group was higher than that in the OGD/R + DEX+ EX527 group (Figure 6a), indicating that TET1 lifted Sirt1 levels. Besides, we performed the MS-PCR experiment to investigate the methylation of the Sirt1 promoter in cardiomyocytes (HCM and AC16). As a result, the Sirt1 promoter in OGD/R group was more methylated than the CON group, and TET1 up-regulation reduced Sirt1 promoter methylation (Figure 6b). In conclusion, TET1 up-regulated Sirt1 by demethylating the Sirt1 promoter.

TET1 overexpression plasmids were transfected into cardiomyocytes (HCM and AC16), and then OGD/R-induced cardiomyocytes were processed with 5 μM DEX for 48 hours, followed by the addition of a Sirt1 inhibitor (EX527). A: The levels of TET1, Sirt1, and NF-κB in AC16 and HCM cells were determined by WB. B: AC16 and HCM cells were treated with OGD/R. MSPCR was adopted for checking the Sirt1 promoter methylation in AC16 and HCM. Data were expressed as mean ±SD, n = 3; **P < 0.01, ***P < 0.001 (vs. OGD/R).NS *P* > 0.05, &&*P* < 0.01 (vs. OGD/R+ DEX). #*P* < 0.05, ##*P* < 0.01, ###*P* < 0.001 (vs. OGD/R+ DEX+EX527).

### 3.7 Knockdown of TET1 abrogated DEX-mediated myocardial protection

**Figure 7.**
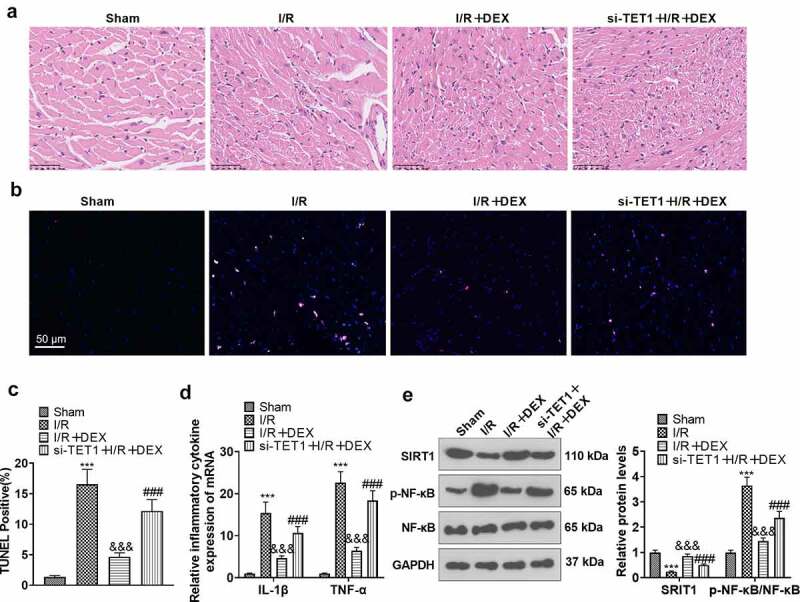
Knockdown of TET1 weakened DEX-mediated myocardial protection.

To further validate the function of TET1/ Sirt1 in the improvement of myocardial H/R injury by DEX, we constructed a rat I/R model. The rats were distributed randomly into Sham, DEX, I/R, I/R + DEX, and si-TET1 + I/R + DEX groups. Rats in the si-TET1 + I/R + DEX group were given si-TET1 lentivirus via tail vein injection prior to surgery. H&E staining displayed that DEX alone did not affect the pathological manifestation of myocardial tissues in rats as compared to the Sham group, but it markedly damaged the myocardial tissues of I/R rats. Nevertheless, DEX treatment greatly ameliorated the pathological damage of myocardial tissues of I/R rats. In parallel, the damage to myocardial tissue was significantly less in the I/R + DEX group than in the si-TET1 + I/R + DEX group (Figure 7a). As demonstrated by TUNEL, compared with the Sham group, myocardial tissue apoptosis was diminished in the DEX group and augmented in the I/R rats, and DEX treatment achieved a significant reduction in myocardial tissue apoptosis in the I/R rats. Meanwhile, there was an increase in apoptosis in myocardial tissues of rats in the si-TET1 + I/R + DEX group versus the I/R + DEX group (Figure 7b). The expression of inflammatory factors IL-1β and TNF-α in rat myocardial tissues was checked by RT-PCR. As a result, the expression of IL-1β and TNF-α was not significantly altered in myocardial tissues of DEX rats versus the Sham group. In contrast, the expression of IL-1β and TNF-α was raised in the myocardial tissues of I/R rats, and the expression of inflammatory factors was distinctly abated in myocardial tissues of I/R rats after DEX treatment. Meanwhile, the expression of inflammatory factors in the myocardial tissues of rats in the I/R + DEX group was considerably lower than that in the si-TET1 + I/R + DEX group (Figure 7c). WB outcomes exhibited that the application of DEX up-regulated Sirt1 and abated NF-κB phosphorylation in rat myocardial tissues and diminished Sirt1 expression and enhanced NF-κB phosphorylation in myocardial tissues of I/R rats versus the Sham group. In contrast, DEX treatment up-regulated Sirt1 and weakened NF-κB phosphorylation in myocardial tissues of I/R rats compared to the I/R group. In parallel, knockdown of TET1 restrained Sirt1 expression and uplifted NF-κB phosphorylation in myocardial tissues of I/R rats versus the I/R + DEX group (Figure 7d). Hence, DEX ameliorated MI/R injury in rats, and knockdown of TET1 attenuated the DEX-mediated cardioprotective effects.

The rat I/R model was established, and the rats were randomized into Sham, DEX, I/R, I/R + DEX, and si-TET1 + I/R + DEX groups. A. H&E staining was applied to inspect the pathological changes in myocardial tissues of rats. B. TUNEL was employed to gauge apoptosis in rat myocardial tissues. C. The expression of inflammatory factors IL-1β and TNF-α in rat myocardial tissues was tested by RT-PCR. D. The expression of Sirt1 and NF-κB in rat myocardial tissues was monitored by WB. ***P < 0.001 (vs. Sham). &&&*P* < 0.01 (vs. I/R). ###*P* < 0.001 (vs. I/R+ DEX). Data were expressed as mean ±SD, n = 5.

## 4. Discussion

Related researches have stated that H/R can cause additional damage to ischemic tissues, thus further aggravating the damage of myocardial cells and the amplification of infarct area [41]. The pathological mechanisms of H/R injury are complex and involve multiple factors and signaling pathways, including apoptosis, inflammation, oxidative stress, and DNA methylation, with the inflammatory response being the main cause, which leads to apoptosis and impedes cardiomyocyte viability [4]. Here, we ascertained that DEX attenuates the apoptosis and inflammation induced by H/R injury, and the possible mechanism is that DEX ameliorates H/R-mediated myocardial injury by heightening TET1 demethylation of the Sirt1 promoter to up-regulate Sirt1.

Dexmedetomidine (DEX) is a highly selective α2-adrenergic receptor agonist, which protects against H/R damage and lessens the percentage of apoptotic cells [42]. Reports have claimed that DEX has strong anti-inflammatory properties and is able to reduce I/R-induced cardiomyocyte injury by suppressing inflammatory signaling [43]. Additionally, DEX prevents H/R-induced myocardial injury by improving oxidative stress and apoptosis [33]. DEX eases H/R injury by abating apoptosis via the hypoxia-inducible factor 1α (HIF-1α) signaling [37]. DEX protects cardiomyocytes from H/R-induced necrotic apoptosis by hampering high mobility group box protein 1 (HMGB1)-mediated inflammations [44]. It is clear from the above study that DEX impedes H/R-induced myocardial injury, which is consistent with our findings. Namely, DEX eases OGD/R-mediated myocardial injury, enhances cardiomyocyte viability, lowers the expression of pro-apoptotic proteins (Bax, Bad and Caspase3, Caspase8, and Caspase9), facilitates the expression of anti-apoptotic proteins (Bcl2) and impedes the profiles of inflammatory factors (TNF-α and IL-1β).

The pathological mechanism of H/R injury is implicated in DNA methylation, and aberrant DNA methylation patterns have been identified in I/R-related organ injury [23]. Related studies have demonstrated that perinatal nicotine exposure aggravates I/R-mediated left ventricular injury and hinders post-ischemic recovery of left ventricular function and coronary flow rate, which is associated with increased DNA methylation [45]. TET1 is a demethylating enzyme that lowers DNA methylation levels. Through demethylation of purinergic receptor P2X 7 (P2rX7), TET1 controls the release of bone marrow mesenchymal stem cell (BMMSC)-derived exosomes and maintains the homeostasis of BMMSCs and skeleton [46]. Here, we discovered that DEX boosted TET1 expression and restrained OGD/R-mediated DNA methylation in cardiomyocytes, and the expression of DNA methylation-related proteins (DNMT1, DNMT3A, and DNMT3B) was notably choked. Knocking down TET1 weakened the cardioprotective effect mediated by DEX, suggesting that TET1mitigated H/R injury by DEX.

Sirt1 is a class of histone deacetylases whose activation is involved in various vital metabolic and physiological processes, including stress resistance, metabolism, apoptosis, and energy homeostasis in ischemic injury and cardiometabolic diseases [47]. As an example, resveratrol impedes the expression of inflammatory factors by activating Sirt1, exhibiting anti-inflammatory effects in cardiac myocytes [48]. Also, Sirt1 contributes to ameliorating the H/R damage. Several reports have stated that microRNA-494 (miR-494) targets Sirt1 to modulate the PI3K/AKT/mTOR pathway, thereby attenuating apoptosis and autophagy and protecting cardiomyocytes from I/R injury [49]. Long non-coding RNA MIAT (lncRNA MIAT) binding to microRNA-330-5p (miR-330-5p) enhances lipopolysaccharide (LPS)-mediated inflammations and oxidative stress in septic cardiomyopathy via NF-κB activation [50]. Absent in melanoma 2 (AIM2) restrains the acetylation of NF-κB by interacting with signal transducer and activator of transcription 1 (STAT1) phosphorylation, thereby reducing the transcription of inflammatory factors in cardiac myocytes [51]. In addition, Sirt1-mediated NF-κB deacetylation hinders the myosin light-chain kinase/myosin light chain-2 (MLCK/MLC2) pathway and endothelin-1 (ET-1) expression, thus alleviating coronary artery spasm [52]. Pterostilbene (PTE) abates NF-κB acetylation in BV-2 microglia through up-regulation of Sirt1, thereby choking the expression of inflammatory factors [53]. Caffeic acid o-nitro phenethyl ester (CAPE-oNO2) attenuates H/R damage on cardiomyocytes by up-regulating Sirt1 and inactivating NF-κB [54]. Other studies have illustrated that a slight increase in local DNA methylation hinders Sirt1 expression in white muscles [55]. In this study, we noticed that DEX facilitated Sirt1 expression, and inhibition of Sirt1 weakened the myocardial protective effect mediated by DEX. Meanwhile, TET1 up-regulated Sirt1 and TET1 boosted the demethylation of the Sirt1 promoter. In conclusion, TET1 up-regulates Sirt1 expression by demethylating the Sirt1 promoter, thus alleviating H/R-induced myocardial injury.

## 5. Conclusion

Overall, our study displays that DEX up-regulates Sirt1 by boosting TET1 demethylation of the Sirt1 promoter, thereby easing H/R-mediated myocardial injury. This paper investigates the mechanism of TET1/Sirt1 in the amelioration of H/R injury by DEX and provides new therapeutic ideas to mitigate H/R injury.

## Data Availability

The data sets used and analyzed during the current study are available from the corresponding author on reasonable request.

## Abbreviation

5-mC: 5-methylcytosine; AIM2: absent in melanoma 2; AMI: Acute myocardial infarction; BMMSC: bone marrow mesenchymal stem cell; CAPE-oNO2: caffeic acid o-nitro phenethyl ester; CCK-8: cell counting kit-8; CHOP: C/EBP-homologous protein; DEX: Dexmedetomidine; DMEM: Dulbecco’s Modified Eagle’s medium; DNMT1: DNA methyltransferase 1; DNMT3A: DNA methyltransferase 3 alpha; DNMT3B:DNA methyltransferase 3 beta; ELISA: Enzyme-linked immunosorbent assay; ET-1: endothelin-1; FBS: fetal bovine serum; GAPDH: glyceraldehyde-3-phosphate dehydrogenase; GPR30: G-Protein Receptor 30; H/R: hypoxia/reoxygenation; HIF-1α: Hypoxia-inducible factor 1α; HMGB1: High mobility group box protein 1; HRP: horseradish peroxidase; IL-1β: interleukin 1β; lncRNA: Long Non-coding RNA LPS: Lipopolysaccharide; miR: microRNA; MLCK/MLC2: myosin light-chain kinase/myosin light chain-2; MS-PCR: methylation-sensitive polymerase-chain reaction; mTOR: mechanistic target of rapamycin; NF-κB: Nuclear factor kappa B; OD: optical delnsity; OGD/R: Oxygen–glucose deprivation and reoxygenation; P2rX7:purinergic receptor P2X 7; p-38 MAPK: p-38 Mitogen-activated protein kinase; PBS: phosphate-buffered saline; PI3K: phosphatidylinositol 3-kinase; PTE: pterostilbene; PVDF: polyvinylidene difluoride; RT-PCR: Reverse transcription-polymerase chain reaction; Sirt1: Silencing information regulator 2 related enzyme 1; STAT1: signal transducer and activator of transcription 1; TBST: Tris-buffered saline with Tween-20; TET1:tet methylcytosine dioxygenase 1; TNF-α:tumor necrosis factor-α.
